# *Clostridioides difficile* Infection in the United States of America—A Comparative Event Risk Analysis of Patients Treated with Fidaxomicin vs. Vancomycin Across 67 Large Healthcare Providers

**DOI:** 10.3390/idr17040087

**Published:** 2025-07-23

**Authors:** Sebastian M. Wingen-Heimann, Christoph Lübbert, Davide Fiore Bavaro, Sina M. Hopff

**Affiliations:** 1 Cologne Excellence Cluster on Cellular Stress Responses in Aging-Associated Diseases (CECAD), Institute of Translational Research, Faculty of Medicine, University Hospital Cologne, University of Cologne, 50931 Cologne, Germany; 2 Department I of Internal Medicine, Center for Integrated Oncology Aachen Bonn Cologne Duesseldorf (CIO ABCD) and Excellence Center for Medical Mycology (ECMM), University Hospital Cologne, Faculty of Medicine, University of Cologne, 50937 Cologne, Germany; 3 Department of Health and Social Affairs, FOM University of Applied Sciences, 50931 Cologne, Germany; 4 German Centre for Infection Research (DZIF), Partner Site Bonn-Cologne, 50931 Cologne, Germany; 5 Department of Infectious Diseases and Tropical Medicine, Hospital St. Georg, 04129 Leipzig, Germany; christoph.luebbert@medizin.uni-leipzig.de; 6 Division of Infectious Diseases and Tropical Medicine, Department of Medicine I, Leipzig University Medical Center, 04103 Leipzig, Germany; 7 Interdisciplinary Center for Infectious Diseases, Leipzig University Medical Center, 04103 Leipzig, Germany; 8 Department of Biomedical Sciences, Humanitas University, Via Rita Levi Montalcini 4, 20090 Milan, Italy; 9 Infectious Diseases Unit—IRCCS Humanitas Research Hospital, Via Manzoni 56, 20089 Milan, Italy; 10 Division of Infectious Diseases, Department I of Internal Medicine, Faculty of Medicine and University Hospital Cologne, University of Cologne, 50937 Cologne, Germany; sina.hopff@uk-koeln.de

**Keywords:** sepsis, candidiasis, survival rate, patient outcome, concomitant diseases

## Abstract

Background/Objectives: *Clostridioides difficile* infection (CDI) is a major cause of infectious diarrhea in the inpatient and community setting. Real-world data outside the strict environment of randomized controlled trials (RCTs) are needed to improve the quality of evidence. The aim of this study was to compare different clinical outcomes of CDI patients treated with fidaxomicin with those treated with vancomycin using a representative patient population in the United States of America (USA). Methods: Comprehensive real-world data were analyzed for this retrospective observational study, provided by the TriNetX database, an international research network with electronic health records from multiple USA healthcare providers. This includes in- and outpatients treated with fidaxomicin (FDX) or vancomycin (VAN) for CDI between 01/2013 and 12/2023. The following cohorts were compared: (i) patients treated with fidaxomicin within 10 days following CDI diagnosis (FDX group) vs. (ii) patients treated with vancomycin within 10 days following CDI diagnosis (VAN group). Outcomes analysis between the two cohorts was performed after propensity score matching and included event risk and Kaplan–Meier survival analyses for the following concomitant diseases/events occurring during an observational period of 12 months following CDI diagnosis: death, sepsis, candidiasis, infections caused by vancomycin-resistant enterococci, inflammatory bowel disease, cardiovascular disease, psychological disease, central line-associated blood stream infection, surgical site infection, and ventilator-associated pneumonia. Results: Following propensity score matching, 2170 patients were included in the FDX group and VAN groups, respectively. The event risk analysis demonstrated improved outcomes of patients treated with FDX compared to VAN in 6 out of the 10 events that were analyzed. The highest risk ratio (RR) and odds ratio (OR) were found for sepsis (RR: 3.409; OR: 3.635), candidiasis (RR: 2.347; OR: 2.431), and death (RR: 1.710; OR: 1.811). The Kaplan–Meier survival analysis showed an overall survival rate until the end of the 12-month observational period of 87.06% in the FDX group and 78.49% in the VAN group (log-rank *p* < 0.001). Conclusions: Our comparative event risk analysis demonstrated improved outcomes for patients treated with FDX compared to VAN in most of the observed events and underlines the results of previously conducted RCTs, highlighting the beneficial role of FDX compared to VAN. Further big data analyses from other industrialized countries are needed for comparison with our observations.

## 1. Introduction

*Clostridioides difficile* infection (CDI) is the leading cause of hospital-acquired diarrhea and infection in the United States of America (USA) [1]. Fortunately, the US Centers for Disease Control and Prevention (CDC) reported a nationwide decline in 2019 with 202,600 cases of hospitalized CDI patients resulting in 11,500 deaths, while case numbers were still higher in 2017 with 223,900 estimated cases among hospitalized patients and 12,800 deaths [2]. More up-to-date epidemiological data from the CDC is not yet available due to delays caused by the COVID-19 pandemic but is expected in 2025. Nevertheless, CDI still poses a significant healthcare burden, leading to substantial clinical, social, and health economic consequences [3]. In addition to strict adherence to infection prevention and control measures and the ongoing implementation of inpatient antibiotic stewardship programs, the decline in CDI cases is attributed to improvements in CDI therapy, leading to fewer recurrent CDI infections [4].

In 2021, the Infectious Diseases Society of America (IDSA) and the Society for Healthcare Epidemiology of America (SHEA) published an updated guideline on the management of CDI in adults [5]. For patients with a first CDI and with a first CDI recurrence, fidaxomicin (FDX) is recommended as first-line therapy, while vancomycin (VAN) is an alternative treatment option if FDX is not available. For patients with multiple CDI recurrences, FDX (with the possibility of an extended therapy regimen), a tapered-pulsed VAN regimen, VAN followed by rifaximin, and fecal microbiota transplantation (FMT) are described as treatment options, with the best data for FMT. By now, the beneficial role of FDX for treating patients with CDI has been examined in randomized controlled trials and mostly single-center, real-world studies [4,6,7,8,9,10,11,12]. The mentioned studies primarily focused on evaluating the efficacy and safety of FDX in comparison to VAN and metronidazole. However, results on detailed clinical outcomes of CDI patients treated with FDX versus VAN remain limited. Therefore, the aim of this study was to compare concomitant clinical events of CDI patients treated with FDX with those treated with VAN using a representative patient population in the USA.

## 2. Material and Methods

### 2.1. Study Design and Data Source

This is a retrospective observational study of patients with CDI who received an inpatient or outpatient treatment across 67 large healthcare providers (e.g., hospitals or rehabilitation centers) in the USA. The overall observational period was from January 2013 to December 2023, and patients coded with the ICD-10-CM code A04.7 (enterocolitis due to *Clostridioides difficile*) and who received targeted CDI treatment with FDX or VAN were included in the analysis. All de-identified patient data were extracted from the TriNetX database (Cambridge, MA, USA), a large collaborative network in the USA comprising data of approximately 118 million patients [13]. The final run of data extraction from the TriNetX database was performed on 9 December 2024. Due to the aggregated data of the TriNetX database, no information on CDI diagnosis by stool culture or the bacteria’s DNA or toxins was available.

### 2.2. Definitions

Two groups were defined within the cohort. The first group included patients who were treated with fidaxomicin (FDX group); the second group included patients who were treated with vancomycin (VAN group). For both groups, targeted CDI treatment started within 10 days following CDI diagnosis, and no other CDI medication was given prior to FDX or VAN within the observed period, which started on the day of the first CDI diagnosis (day 0). The patient-specific observational period for the event risk and Kaplan–Meier survival analyses was from day 0 to day 365.

Based on the current evidence, our event risk and Kaplan–Meier survival analyses included the following 10 events which occurred during the observational period: (i) death, (ii) sepsis (ICD-10-CM codes: A41., R65.2, A40.), (iii) candidiasis (ICD-10-CM codes: B37), (iv) infection with vancomycin-resistant enterococci (VRE) (ICD-10-CM codes: B95.2, Z16.21, A49.8, R89), (v) inflammatory bowel disease (IBD) (ICD-10-CM codes: K52.8, K50., K51., K90), (vi) cardiovascular disease (ICD-10-CM codes: I10., I10.-I1A, I10.-I15., I20.-I25., I26.-I28. I30.-I5A, I30.-I52., I60.-I69., I70.-I79., I80.-I89., I95.-I99), (vii) psychological disease (ICD-10-CM codes: F31., F32., F33., F40.-F48., F20), (viii) central line-associated blood stream infection (CLABSI) (ICD-10-CM code: T80.211), (ix) surgical site infection (ICD-10-CM codes: T81.42, T81.43, T81.44, T81.49), and (x) ventilator-associated pneumonia (VAP) (ICD-10-CM code: J95.851). To ensure a well-balanced group distribution, a propensity score matching based on age and gender was performed to conduct the analysis in a 1:1 ratio. Patients with other gut-related conditions were not excluded from the analyses due to an impactful reduction in the *n*, consequently influencing the statistical power.

### 2.3. Statistical Analysis

Patient demographics (age and gender) before and after propensity score matching included mean values and standard deviation (SD), frequency distribution, percentages, and *p*-values (Student’s *t*-test). For follow-up analysis in days, median and interquartile range (IQR) were used. The event risk analysis included frequency distribution, risk difference, risk ratio (RR), and odds ratio (OR), all including the 95% confidence intervals (CIs). In addition, Kaplan–Meier survival analyses were conducted for all the included events that occurred during the observational period, including hazard ratios (HRs) and 95% CI, as well as log-rank test *p*-values. Our aim was to analyze all-cause mortality, meaning that the Kaplan–Meier analyses do not represent mortality rates due to CDI, which was also not possible due to the aggregated data of the TriNetX database. All statistical analyses were performed on the TriNetX platform, which relies on the built-in statistical software R version 4.0.2 (R Foundation for Statistical Computing, Vienna, Austria).

### 2.4. Ethical Considerations and Informed Consent

Any data displayed on the TriNetX platform in aggregate form, or any patient-level data provided in a dataset generated by the TriNetX platform, only contain de-identified patient data. Therefore, no ethics vote was necessary for this study. In addition, this retrospective study is exempt from informed consent. The data reviewed is a secondary analysis of existing data, does not involve intervention or interaction with human subjects, and is de-identified as per the de-identification standard defined in Section §164.514(a) of the HIPAA Privacy Rule. The process by which the data is de-identified is attested to through a formal determination by a qualified expert, as defined in Section §164.514(b)(1) of the HIPAA Privacy Rule [13].

## 3. Results

Prior to propensity score matching, 134,545 patients who initially received VAN and 2170 patients who initially received FDX were identified. Following the propensity score matching, 2170 patients were included in the FDX group and VAN groups, respectively. Patient characteristics were well-matched based on age and gender (Table 1).

Event risk analysis demonstrated improved outcomes for patients treated with FDX compared to VAN in 6 out of 10 analyzed events. The highest RR and OR for the occurrence of an event were observed for sepsis (RR: 3.409, 95% CI: 2.437–4.767; OR: 3.635, 95% CI: 2.560–5.161), candidiasis (RR: 2.347, 95% CI: 1.683–3.275; OR: 2.431, 95% CI: 1.721–3.434), and death (RR: 1.710, 95% CI: 1.477–1.980; OR: 1.881, 95% CI: 1.585–2.232). Further information on the event risk analyses is presented in Table 2.

**Figure 1:**
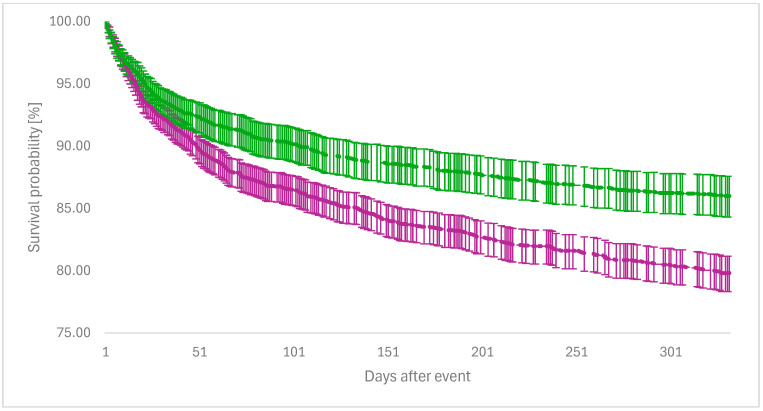
Overall survival at day 365 between the FDX group vs. VAN group. Green line = FDX group; purple line = VAN group; index event = day 0; Hazard ratio = 1.713 (95% CI: 1.462–2.006); Log-Rank test *p*-value ≤ 0.001; Survival probability in the FDX group: 87.06%; Survival probability in the VAN group: 78.49%.

Kaplan–Meier survival analysis showed an overall survival rate until the end of the 12-month observational period of 87.06% in the FDX group and 78.49% in the VAN group (log-rank *p* < 0.001; Figure 1). The use of FDX as primary treatment for CDI had no impact on survival rates for subsequent events: surgical site infection (log-rank *p* = 0.988), IBD (log-rank *p* = 0.889), and VAP (log-rank *p* = 0.252). For all other events occurring during the observational period, the use of FDX was superior to VAN with respect to the survival rates. A comprehensive overview of the values of the Kaplan–Meier survival analysis is given in Table 3.

Based on the official definitions of regions in the USA by United States Census Bureau [14], the geographic distribution of patients in the FDX vs. VAN group was as follows: northeast (24% vs. 26%), midwest (19% vs. 21%), south (35% vs. 35%), west (20% vs. 16%), unknown (1% vs. 2%).

## 4. Discussion

Our comparative event risk analysis demonstrated improved outcomes of patients treated with FDX compared to VAN in most of the observed events during the observational period. We used a 12-month observational period for the event risk and Kaplan–Meier analyses to prevent a time-dependent bias of events that are unlikely to be associated with the occurrence of CDI or its treatment. The highest RR and OR were found for sepsis (RR: 3.409; OR: 3.635), candidiasis (RR: 2.347; OR: 2.431), and death (RR: 1.710; OR: 1.811). The clinical relevance of these comorbidities is in line with previously published data. A multicenter study from Mexico demonstrated septic shock and abdominal sepsis as a major risk factor for the death of patients with CDI [15]. A further large real-world data analysis based on healthcare claims from the USA highlighted sepsis as the leading clinical complication within a 12-month observational period following CDI diagnosis, especially for patients with recurrent CDI [16]. The study also underlined the substantial healthcare burden of CDI patients with other life-threatening comorbidities. Two multicenter studies from Italy and the USA analyzed the impact of nosocomial BSI and candidemia secondary to CDI and demonstrated a significant higher rate of mortality and admission to an intensive care unit compared to controls [17,18]. Nevertheless, none of the studies analyzed the impact of specific antibiotic treatment for CDI on sepsis and candidemia. However, it should be noted that several studies have described disruption of the gut microbiota and/or a cellular inflammatory response resulting from impaired gut barrier function and immune response to CDI toxins as pathophysiological causes of sepsis and CLABSI in patients with CDI [19,20]. In addition to this evidence, it should be noted that several studies have demonstrated the beneficial role of FDX in preventing major changes in the intestinal microbiome composition and reducing toxin production [21,22].

One of the major strengths of our study is the large real-world data sample size across 67 healthcare providers. However, caution is needed in the interpretation of the study results, particularly due to the positive risk/odds/hazard ratios of patients treated with FDX compared to VAN. First, FDX was approved by the FDA in 05/2011 and our observational period started in 01/2013. We assume that, especially in the first years of the observational period, CDI patients were still predominantly treated with VAN in line with guideline recommendations, so that a time bias cannot be ruled out. The further development of medical progress over the observational period should also be taken into account. Unfortunately, it was not possible to evaluate the data with regard to a comparison of prescription numbers of FDX and VAN over the years. Second, a recently published study demonstrated that higher overall wealth is associated with lower mortality, higher income, and access to healthcare insurance in the USA [23]. In addition, privately insured patients also have the option of receiving FDX at a reduced out-of-pocket payment. It can be assumed that lower-income patients have limited opportunities to obtain FDX for CDI due to skewed access to medicines, particularly in the outpatient healthcare sector. Consequently, we cannot exclude the possibility that a selection and/or population bias may have influenced our results, and it was unfortunately not possible to evaluate the data based on, e.g., health insurance status. Third, we cannot exclude the possibility that large outbreaks with the highly virulent strain 027 might have influenced the epidemiology and outcomes of CDI as well as the presented results. Fourth, we cannot guarantee accurate ICD-10-CM coding by the personnel of the 67 healthcare providers included in this study. Additionally, it was outside the scope of this study to analyze specific patient cohorts, e.g., cancer patients or those who were immunosuppressed. We do not assume that a bias was created, as the use of FDX or VAN is in general independent of the underlying disease. Apart from the above limitations, our results highlight the potential of FDX to improve patient outcomes and reduce severe concomitant diseases. Further big data analyses from other industrialized countries are needed for comparison with our observations, e.g., in Germany where another study by Lübbert et al. has analyzed secondary health claims data of patients with CDI based on ICD-10 coding [24].

## Figures and Tables

**Table 1:** Patient demographics before and after propensity score matching.

Cohort (Before PSM)	Characteristic	Mean ± SD	Patients (*n*)	% of Cohort	*p*-Value
VAN group	Age at day 0	59.8 ± 20.5	134,545	100%	<0.001
FDX group		57.5 ± 20.1	2170	100%	
VAN group	Female gender	n.a.	70,361	52.3%	<0.001
FDX group		n.a.	1353	62.4	
**Cohort (after PSM)**					
VAN group	Age at day 0	57.5 ± 20.1	2170	100%	1
FDX group		57.5 ± 20.1	2170	100%	
VAN group	Female gender	n.a.	1353	62.4%	1
FDX group		n.a.	1353	62.4%	

FDX = fidaxomicin; n.a. = not applicable; PSM = propensity score matching; SD = standard deviation; VAN = vancomycin.

**Table 2:** Event risk analysis.

Event	Event Occurred in FDX Group (Patients with Event/Overall Patients; n)	Event Occurred in VAN Group (Patients with Event/Overall Patients; n)	Risk Difference (95% CI)	Risk Difference *p*-Value	Risk Ratio (95% CI)	Odds Ratio (95% CI)
Candidiasis	49/1963	105/1792	0.034 (0.021–0.047)	<0.001	2.347 (1.683–3.275)	2.431 (1.721–3.434)
Cardiovascular disease	76/715	64/379	0.063 (0.019–0.107)	0.003	1.589 (1.167–2.162)	1.708 (1.193–2.446)
CLABSI	10/2161	18/2129	0.004 (−0.001–0.009)	0.120	1.827 (0.845–3.949)	1.834 (0.845–3.983)
Death	244/2156	415/2144	0.080 (0.059–0.102)	<0.001	1.710 (1.477–1.980)	1.881 (1.585–2.232)
IBD	69/1646	67/1543	0.002 (−0.013–0.016)	0.834	1.036 (0.745–1.439)	1.037 (0.736–1.463)
Psychological disease	109/1243	136/1146	0.031 (0.007–0.055)	0.013	1.353 (1.066–1.718)	1.401 (1.074–1.828)
Sepsis	46/1826	113/1316	0.061 (0.044–0.077)	<0.001	3.409 (2.437–4.767)	3.635 (2.560–5.161)
Surgical site infection	15/2148	15/2129	0.000 (−0.005–0.005)	0.981	1.009 (0.494–2.059)	1.009 (0.492–2.069)
VAP	10/2166	10/2138	0.000 (−0.004–0.004)	0.977	1.013 (0.423–2.429)	1.013 (0.421–2.439)
VRE infection	75/1997	120/1891	0.026 (0.012–0.040)	<0.001	1.690 (1.275–2.239)	1.736 (1.292–2.334)

CLABSI = central line-associated blood stream infection; CI = confidence interval; FDX = fidaxomicin; IBD = inflammatory bowel disease; VAN = vancomycin; VAP = ventilator-associated pneumonia; VRE= vancomycin-resistant enterococci.

**Table 3:** Kaplan–Meier survival analysis until the end of the observational period.

Event	Survival Probability with Event in the FDX Group (%)	Survival Probability with Event in the VAN Group (%)	Log-Rank Test *p*-Value	Hazard Ratio (95% CI)
Candidiasis	96.82	92.74	<0.001	2.350 (1.674–3.299)
Cardiovascular disease	86.91	80.40	0.012	1.526 (1.094–2.128)
CLABSI	99.87	98.96	<0.001	9.105 (2.113–39.240)
IBD	94.77	94.58	0.889	1.024 (0.732–1.433)
Psychological disease	88.37	84.38	0.017	1.358 (1.055–1.747)
Sepsis	96.93	89.95	<0.001	3.399 (2.413–4.789)
Surgical site infection	99.11	99.13	0.988	0.995 (0.486–2.034)
VAP	99.76	99.51	0.252	1.991 (0.599–6.611)
VRE infection	95.56	92.44	<0.001	1.684 (1.262–2.247)

CLABSI = central line-associated blood stream infection; CI = confidence interval; FDX = fidaxomicin; IBD = inflammatory bowel disease; VAN = vancomycin; VAP = ventilator-associated pneumonia; VRE = vancomycin-resistant enterococci.

## Data Availability

Restrictions apply to the availability of these data, as the funder is the owner of the data.
